# The intensity of organ support: Restrictive or aggressive therapy for critically ill patients

**DOI:** 10.1016/j.jointm.2023.04.002

**Published:** 2023-05-25

**Authors:** Hongxiang Li, Yuting Li, Yao Fu, Xinyu Zhang, Dong Zhang

**Affiliations:** Department of Intensive Care Medicine, The First Hospital of Jilin University, Changchun 130021, Jilin, China

**Keywords:** Critically ill patients, Organ support, Aggressive therapy, Restrictive therapy, Prognosis

## Abstract

The intensity of organ support has received attention in recent years. To make better clinical decisions, we should understand the mechanisms and benefits, and disadvantages of the different intensities of organ support in critically ill patients. Therapeutic strategies such as supplemental oxygen therapy, mechanical ventilation, respiratory stimulant, vasoactive agents, transfusion, albumin infusion, fluid management, renal placement, and nutrition support, if they are implemented in accordance with an aggressive strategy, could result in side effects and/or complications, resulting in iatrogenic harm in critically ill patients. It is found that the intensity of organ support is not a determining factor in prognosis. A normal rather than supernormal physiological target is recommended for support therapy.

## Introduction

**Figure 1:**
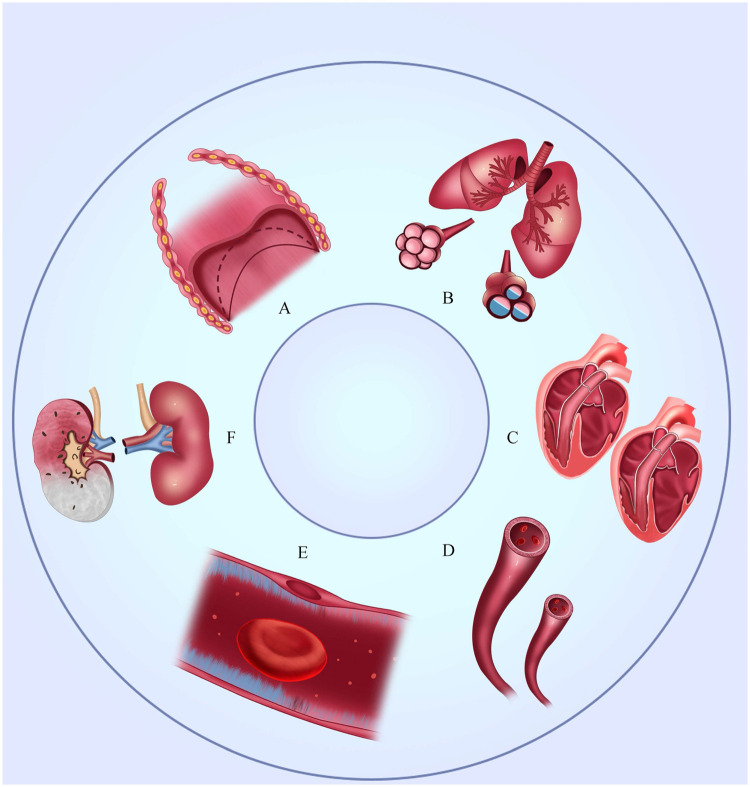
The organ injury in critically ill patients. A: Diaphragmatic fatigue; B: Alveolar edema; C: Cardiac dysfunction; D: Vasoconstriction or vasoparalysis; E: Breakdown of the molecular structure of endothelial glycocalyx; F: Decreased renal function.

Patients with organ dysfunction (sequential organ failure assessment score ≥2) account for more than 80% of those in the intensive care unit (ICU),^[^^1^^]^ and these dysfunctions are usually shown in critically ill patients, for instance, the diaphragmatic fatigue in chronic obstructive pulmonary disease (COPD),^[^^2^^]^ alveolar edema in acute respiratory distress syndrome (ARDS),^[^^3^^]^ cardiac dysfunction in shock,^[^^4^^]^ vasoparalysis and vascular barrier dysfunction in septic shock,^[^^4^^]^ decreased renal function in acute kidney injury (AKI),^[^^5^^]^ and so on (Figure 1). There is no immediate treatment for these injuries, and organ support is one of the main strategies for these critically ill patients, which includes oxygen support, mechanical ventilation (MV), fluid resuscitation, transfusion and albumin infusion, vasopressor application, renal replacement therapy (RRT), and nutrition support. These strategies temporarily support and replace the function of multiorgan systems and provide opportunities and time for managing the primary disorder.

The intensity of organ support has received attention in recent years. Aggressive organ support is usually regarded as one of the basic treatment strategies for patients in the ICU.^[^^6^^]^ However, these medical interventions have side effects and/or complications. Additionally, the physiology of the critically ill patient is often fragile, making them particularly vulnerable to iatrogenic harm.^[^^7^^]^ Advocating for the “less is more” principle has attracted increasing attention because aggressive support causes iatrogenic harm.^[^^8^^]^ To make better clinical decisions, we should understand the mechanisms, benefits, and disadvantages of the different intensities of organ support in critically ill patients.

## Supplemental Oxygen Therapy: High *vs.* Low Oxygenation Targets

Oxygen is essential for maintaining metabolism and life in animals and humans. Hypoxia causes cell metabolism impairment and even cell death. However, oxygen can cause significant side effects although it is a vital “drug” for hypoxic patients.^[^^9^^]^ Excessive supplemental oxygen supply is commonly seen in acute and critically ill patients. Studies have shown that supplemental oxygen potentially produces adverse physiological effects such as increased coronary vascular resistance and reduced coronary blood flow.^[^^10^^]^ Furthermore, oxygen carries the risk of generation of reactive oxygen species, which react with cellular macromolecules and alter their biochemical or physical properties, resulting in cell dysfunction or death.^[^^11^^]^

The targets of oxygen therapy are poorly understood although the importance of supplemental oxygen therapy has been emphasized.^[^^12^^]^ Various clinical studies have revealed the damage of aggressive oxygen support.^[^^13^^,^^14^^]^ A single-center, open-label, randomized clinical trial for critically ill patients with shock, respiratory failure, and renal or liver failure with an ICU duration of no less than 72 h revealed that a conservative protocol for oxygen therapy maintaining an arterial partial pressure of oxygen 70–100 mmHg or arterial oxygen saturation (SaO_2_) 94%–98% resulted in lower ICU mortality compared to conventional therapy (allowing arterial partial pressure of oxygen up to 150 mmHg or SaO_2_ values 97%–100%).^[^^15^^]^ In a pragmatic, cluster-randomized, cluster-crossover trial conducted in the emergency department and medical ICU, there were no differences in mortality among groups of patients in which a lower, intermediate, or higher peripheral oxygen saturation target (90%, 94%, and 98%) was used,^[^^16^^]^ which shows the important implication that the low limit of normal physiologic target achieved by organ support is just only enough.

The guidelines for oxygen therapy recommend a target SaO_2_ range of 90%–94% for acutely ill patients using a minimum amount of oxygen and 88%–92% for those at risk of hypercapnic respiratory failure. It is noteworthy that only patients with carbon monoxide poisoning, cluster headaches, sickle cell crisis, or pneumothorax might benefit from SaO**_2_** approaching 100%.^[^^17^^]^

## Respiratory Stimulant: Strengthening *vs.* Resting Respiratory Muscles

In patients with COPD, diminished gas exchange efficiency of the lung leads to increased respiratory drive and ventilatory requirements. However, the work of breathing in severe COPD is 10 times greater than normal due to increased inspiratory resistance, dynamic hyperinflation, and gas exchange inefficiency.^[^^18^^]^ Respiratory muscle fatigue easily develops while ventilation must be maintained at a relatively high level.^[^^2^^]^ In the setting of acute exacerbation of COPD, hypercapnic respiratory failure occurs when respiratory muscles no longer provide sufficient ventilation, meaning that reserve function or compensatory capacity burns out.^[^^18^^]^ At this time, using a respiratory stimulant to increase ventilation by strengthening respiratory muscles is not a wise choice, as was proven in a previous trial.^[^^19^^]^ In summary, respiratory stimulants are not recommended for COPD patients with respiratory failure, while MV is recommended for ventilatory support.^[^^20^^]^

## MV: the Normal *vs.* Lower Tidal Volume

MV is a key treatment strategy that is associated with several physiological derangements including increased pulmonary venous admixture and physiological dead space and decreased respiratory system compliance in patients with ARDS, which is a condition of diffuse alveolar injury caused by pneumonia, aspiration, or sepsis.^[^^3^^]^ Large tidal volumes would cause lung injury in ARDS with a collapsed lung, decreased volume of aerated lung, and decreased lung compliance.^[^^21^^]^ Even normal tidal volumes delivered with airway pressures, which are considered to be safe for the uninjured lung, may cause regional overdistention because the volume of the aerated lung is reduced in ARDS patients.^[^^22^^]^ Ventilation with a lower tidal volume protects the lungs from excessive stretch and results in decreased mortality and ventilation-free time.^[^^23^^]^ So, MV with low tidal volume is a perfect example of a restrictive therapy according to the pathophysiological mechanism of ARDS.

## Vasoactive Agents: the High *vs.* Low MAP

Besides fluid resuscitation, the application of vasoactive agents is important for maintaining hemodynamic stability and achieving targeting microcirculation in shock. However, vasoactive medications are symptomatic and supportive treatment but not etiological treatment and may decrease tissue blood flow and increase the risk of myocardial ischemia.^[^^4^^]^

Septic shock, identified as persisting hypotension requiring vasopressors to maintain mean arterial pressure (MAP) ≥65 mmHg,^[^^24^^]^ is commonly experienced in patients admitted to the ICU.^[^^4^^]^ Abnormal vasodilatation and relative hypovolemia are the basic pathophysiological alterations in septic shock. Vasopressors and fluid therapy effectively counter pathophysiological changes in septic shock.^[^^4^^]^ However, high blood pressure achieved with a vasopressor is detrimental for patients with septic shock. A high MAP (80–85 mmHg) compared with a low MAP (65–70 mmHg) maintained with vasopressors cannot improve mortality, on the contrary, the incidence of atrial fibrillation is higher in the high MAP group.^[^^25^^]^

Inotropic agents applied to compensate for insufficient cardiac output due to non-cardiac pathophysiological mechanisms such as septic shock should be controlled. Early goal-directed therapy^[^^26^^]^ guiding early hemodynamic resuscitation with dobutamine to improve oxygen delivery cannot actually decrease mortality in septic shock.^[^^27^^]^ Levosimendan increases myocardial contraction with a minimal increase in oxygen demand and does not impair diastolic relaxation.^[^^28^^]^ However, levosimendan application in adults with sepsis is not associated with less severe organ dysfunction or lower mortality. Instead, it is linked to a lower likelihood of successful weaning from MV and a higher risk of supraventricular tachyarrhythmia.^[^^29^^]^ Therefore, for most patients with septic shock, inotropic agents may not be an effective solution.

Inotropes are usually indicated in patients with acute heart failure in the presence of peripheral hypoperfusion due to low cardiac output and cardiac shock. However, there is no evidence that inotropes can improve mortality in patients with cardiac shock.^[30]^ It is recommended that inotropes be used at the lowest dose and for the shortest time.^[^^31^^]^

## Fluid Management: the Restrictive *vs.* Aggressive Fluid Resuscitation

Fluid therapy is essential in the management of shock because it increases circulating blood volume and cardiac output.^[^^4^^]^ Excessive fluid volume in circulation is harmful. In a clinical setting, aggressive fluid resuscitation leads to pulmonary edema, high intra-abdominal pressure, and hemodilution, resulting in decreased delivery of oxygen ^[^^32^^]^ and increased risk of mortality.^[^^33^^]^ Increased cumulative fluid in the early stage of septic shock is associated with increased mortality.^[^^34^^]^

It is believed that restricting volumes of resuscitation fluid helps prevent adverse medical events from worsening in patients with septic shock.^[^^35^^,^^36^^]^ But, two large randomized controlled trials (RCTs) show the early or continuous restriction of intravenous fluid does not result in a change of mortality among patients with sepsis-induced hypotension or septic shock.^[^^37^^,^^38^^]^ Like supplemental oxygen therapy, “dose” is not the main focus within a certain range, and it is only just enough.

## Transfusion and Albumin Infusion: the Restrictive *vs.* Liberal Transfusion Strategy

An early RCT found that a restrictive red blood cell transfusion strategy for critically ill patients was at least as effective as or possibly superior to a liberal transfusion strategy with the target hemoglobin concentration of 10.0 g/dL, with the exception of acute myocardial infarction and unstable angina.^[^^39^^]^ Even in patients with septic shock, a restrictive red blood cell transfusion strategy is also applicable.^[^^40^^]^ Moreover, compared with a liberal transfusion strategy, a restrictive strategy significantly improves outcomes in patients with acute upper gastrointestinal bleeding.^[^^41^^]^ It is of note that a restrictive red blood cell transfusion strategy is not recommended for patients with acute myocardial infarction and unstable angina, after cardiac surgery^[^^40^^]^ or with brain injury.^[^^42^^]^

Hypoalbuminemia (generally defined as a serum albumin concentration ≤30 g/L) is common in critically ill patients. It is usually caused by direct loss and redistribution from the intravascular to the extravascular space due to increased capillary permeability.^[^^43^^]^ Recent studies have confirmed that not only albumin but also endothelial glycocalyx plays a key role in maintaining colloid osmotic pressure.^[^^44^^]^ These two substances form the endothelial surface layer together to maintain vascular barrier function.^[^^43^^]^ Experiments have shown that around 10 g/L albumin is sufficient to generate a functional endothelial surface layer.^[^^45^^]^ Vascular barrier dysfunction is probably not due to hypoalbuminemia but rather to a breakdown of the molecular structure of endothelial glycocalyx because of hypervolemia, ischemia/reperfusion injury, or systemic inflammation.^[^^43^^]^ At present, there is no evidence that albumin is superior to crystalloid for intravascular volume resuscitation in critically ill patients.^[^^46^^,^^47^^]^

## Renal Placement Therapy: the Early *vs.* Delayed RRT Initiation

AKI is defined as acute changes in renal function without specific effective pharmacologic therapy for AKI to date. Supportive treatment with RRT is a routine strategy for AKI patients.^[^^48^^]^ Like other supportive therapies, aggressive therapy with RRT has not been proven to improve the mortality of patients with AKI. At first, the higher dose of RRT (>20–25 mL/kg/h) could not reduce mortality and enhance kidney function recovery.^[^^5^^]^ Then, the trial proved that except for current conditions such as immediate life-threatening complications, early RRT initiation for AKI could not bring clinical benefit. In contrast, the recovery of renal function was more rapid, and catheter-related infections occurred less frequently in patients with the delayed-strategy RRT than that in the early strategy.^[^^49^^]^ A previous multicenter RCT also found that the overall 90-day mortality did not differ between septic shock patients with severe AKI who received an early RRT with a median of 7.6 h and those who had a delayed strategy with a median of 51.5 h.^[^^50^^]^

## Nutrition Support: the Early Restrictive *vs.* Aggressive Nutrition Support

Sufficient and appropriate nutrition is essential for sustaining the body's metabolism. How to perform nutrition support because of the altered metabolism in the acute phase of critical illness is a challenging issue.^[^^51^^]^ The acute phase of the critical illness usually lasts for approximately 7 days, including an early period (days 1–2) defined by metabolic instability and a large increase in catabolism and a late period (days 3–7) defined by a significant muscle waste and a stabilization of the metabolic disturbances.^[^^51^^]^

These sequential metabolic changes are complex and cannot be fully inhibited by exogenous caloric supply.^[^^52^^]^ In the acute phase following a severe insult, aggressive nutritional therapy may be detrimental as it may cause a metabolic overload and/or suppress the ubiquitin–proteasome pathway and related autophagy pathway, which are potentially important for cellular repair and organ recovery.^[^^53^^]^ In addition, critically ill patients often suffer from gastrointestinal dysfunction due to the vulnerable features of the intestine. Early and aggressive enteral nutrition may be contraindicated in patients with acute gastrointestinal injury, and adverse prognostic effects have been validated in critically ill patients with an overloaded gut.^[^^54^^]^ Recently, a prospective cohort study finds that early nutrition support, especially enteral nutrition in the ICU was significantly associated with increased mortality.^[^^55^^]^ Furthermore, an increasing number of studies support hypocaloric nutrition supply for the acute phase of critical illness.^[^[56], [57], [58], [59]^]^ For patients receiving early supplemental parenteral nutrition, the dose rather than the route of delivery (enteral or parenteral nutrition) affects the outcome.^[^^60^^]^ The number of macronutrients administered early may worsen clinical outcomes.^[^^61^^,^^62^^]^

## Conclusions

Aggressive therapy is of no benefit to critically ill patients. It is against normal pathophysiological changes and brings side effects. It may not be associated with survival benefits, especially when the primary disease is incurable. As the specific therapeutic goal maintained by organ support is crossing the threshold, the intensity of organ support will not significantly impact the prognosis in critically ill patients.

## Author Contributions

DZ and HL drafted the manuscript. HL and XZ made the figure. YF revised the manuscript. All authors have reviewed and approved the final draft of the manuscript.

## Acknowledgments

None.

## Funding

This work was supported by the Department of Finance in Jilin Province (grant number: JLSWSRCZX2021-070).

## Ethics Statement

Not applicable.

## Conflict of Interest

The authors declare that they have no known competing financial interests or personal relationships that could have appeared to influence the work reported in this paper.

## Data Availability

The data sets generated during and/or analyzed during the current study are available from the corresponding author upon reasonable request.
